# Fear of COVID-19, Stress and Coping Strategies among Nurses during the COVID-19 Pandemic’s Second Wave: A Quasi-Intervention Study

**DOI:** 10.2174/18740179-v18-e221221-2022-2

**Published:** 2023-12-21

**Authors:** Doaa I. Omar, Samar A. Amer, Abeer E. Abdelmaksoud

**Affiliations:** 1 Department of Community, Environmental and Occupational Medicine, Faculty of Medicine, Benha University, Benha, Egypt; 2 Department of Public Health, and Community Medicine, Faculty of Medicine, Zagazig University, Zagazig, Egypt; 3 Royal Colleague of General Practitioner (INT), London, United Kingdome; 4 Department of Community, Environmental and Occupational Medicine, Faculty of Medicine, Benha University, Benha, Egypt

**Keywords:** Stress, Fear of COVID-19, Coping, Nurses, University hospitals, Pandemic

## Abstract

**Background:**

The COVID-19 pandemic and its related consequences caused a higher risk of mental health problems for nurses. Hence, this study aims to reduce the level of fear and stress related to the COVID-19 pandemic and promote active coping among Egyptian nurses.

**Methods:**

This quasi-intervention study was conducted on 125 nurses working at Benha’s University hospitals, who were selected by a systematic random sampling technique within the time interval of March 2021 to July 2021. The study was conducted using the fear of COVID-19 scale, the stress scale of depression, anxiety and stress scales, and the Brief (COPE) inventory scale.

**Results:**

The mean ages of the studied nurses were 36.70 ± 9.50. Almost half of the studied nurses were males and married. Before the intervention, 47.2% of nurses had severe stress levels while 82.4% had a high level of fear of COVID-19. Experience years, type of department, and worries about vaccine side effects were the predictors of the fear of COVID-19. A significant difference (p = .000) was found between both mean stress and fear scores pre-intervention (15.27 ± 5.47 and 25.56 ± 6.13) and post-intervention (4.87 ± 2.14 and 11.92 ± 2.43). The most prevalent coping strategies among nurses before the intervention were self-distraction (5.03 ± 1.53), followed by behavioral disengagement and self-blaming. However, after the intervention, religion was found to be the utmost coping mechanism **(**6.12 ± 1.17), followed by positive reframing and acceptance.

**Conclusion:**

The majority of the nurses in the study reported a significant fear of COVID-19, and around half of the nurses had severe stress as a result. After the intervention, the stress and fear scores were reduced by half or even less. Age, longer work experience, and worries about the vaccine were the predictors of fear of COVID-19. The coping strategies used after the intervention shifted toward active coping strategies.

**Clinical Trial Registration Number:** 10-11-008-701

## 1 INTRODUCTION

The Coronavirus 2019 disease (COVID-19) outbreak, caused by the severe acute respiratory syndrome Coronavirus 2 (SARS-CoV-2), was a major public health crisis [1] that was declared by the World Health Organization (WHO) as a global pandemic on March 11, 2020 [2]. On October 21, 2021, there were 241,886,635 confirmed cases of COVID-19 and 4,919,755 deaths worldwide [3]. In Egypt, there have been 321,084 confirmed cases of COVID-19 with 18,105 deaths reported to WHO [4].

Healthcare Workers (HCWs) were at high risk of infection during the COVID-19 pandemic, especially frontline health-care workers, who are directly involved in the sample collec-tion, management, and care of patients during the outbreak. These frontline HCWs were at a higher risk of developing psychological distress and mental health problems [5].

Some of the immediate psychological effects among frontline HCWs are distress, anxiety, depression, and fear of contracting or spreading the infection to family, friends, and colleagues [6]. The COVID-19 pandemic itself as well as the public health measures, *e.g*., lockdown, and their subsequent consequences, such as job losses, financial problems, disruption of daily activities, ongoing restrictions, lack of certainty of returning to usual life, and mortality, were also likely among the reasons of the increase the risk of developing long-term mental health disturbances [7, 8].

Excessive fear of COVID-19, besides the possibility of being infected or infecting others, may worsen anxiety symptoms (which is, by definition, excessive fear and avoidance) (Shin and Liberzon 2010) in people with pre-existing psychological disorders and could cause psychological distress in the general population. Excessive fear of COVID-19 has also been associated with cases of suicide in Bangladesh [9], India [10] and Italy [11].

On the other hand, fear could motivate COVID-19-related behavioral changes. A study found that fear of COVID-19 was a significant predictor of improved hand hygiene and social distancing, implying that fear of COVID-19 plays a key role in compliance with COVID-19-related public health measures [12].

Due to their daily routine of monitoring, managing, and assisting patients severely infectious patients, frontline nurses may be more susceptible to the aforementioned physical and psychosocial hazards pertaining to their job [13]. Nurses’ struggle to maintain their mental and emotional health while caring for these patients can create a professionally and ethically compromising position that some nurses are unable to effectively cope with [14, 15].

Effective stress management in nurses is aimed at limiting and controlling the impact of stressful events on one's physical, social, and emotional functioning, as well as their ability to meet work requirements [15, 16]. According to Downey *et al*. (2011), nurses may manage stressful situations by engaging in useful tasks and realistic workloads [17].

Expressing emotions might lower distress, anxiety, depression, and hostility levels in stressful situations. Different coping styles may be emotion-focused or problem-focused, while others could manifest in active and avoidant coping or maladaptive coping strategies (denial, substance use, and venting of negative emotions), which allow for lowering the experienced stress [18].

Some of the major stress coping strategies adopted by nurses include accepting what cannot be changed, expressing feelings instead of bottling them up, time management, delegation of duties, and slacking off duties. Excessive eating, smoking, and drug and/or alcohol abuse are some of the negative stress coping strategies that could be adopted by them [19].

Stress perception is a subjective changeable phenomenon. Special focus is made on stress coping mechanisms, which determine the positive or negative influence of stress on an individual. There are different stress coping mechanisms deployed, including mobilizing behavioral and cognitive resources to fulfill demands that are subjectively viewed as being beyond personal capabilities [18].

This study aims to assess stress levels and fear of COVID-19 among nurses in Benha University Hospitals, as well as to establish psychological support and stress management programs to minimize stress and promote active coping during the COVID-19 pandemic. Moreover, the study assesses the stress-coping strategies used by the studied nurses, implements well-structured training to reduce stress and fear of COVID-19, enhances active coping, and studies the level of stress and fear of COVID-19 and the used/applied coping techniques before and after the intervention.

## 2 METHODS

### 2.1 Participants and Study Design

During the second wave of the COVID-19 pandemic, this quasi-intervention study was conducted on a randomly selected sample of nurses working in Benha University hospitals to investigate the impact of infection control and training courses to manage stress (which was conducted within the period from March 2021 to July 2021). The selection criteria were based on the following aspects: active nurses at Benha University hospitals during the study period (who were working for at least one year), and their consent to participate in the study.

### 2.2 Sample Size and Technique

Based on the prevalence of stress among nurses in a recent research study, which was 91.7% [20], the sample size was estimated using an online sample size calculator. Based on a 5% precision, an 80% test power, and a 95% confidence level, the estimated sample size was 105 participants. About a 20% increase in the sample took place, accounting for dropout or attrition of the sample.

A systematic random sampling technique was used. Of the total 1,500 nurses, only 990 fulfilled the selection criteria as recorded in the hospital registries. For every eight nurses, a nurse was selected to participate in the study, reaching 125 nurses, representing the following departments: 11 from care units, 20 from the emergency department, 20 from medical departments, 63 from the pediatric department, and 11 from other departments.

### 2.3 Study Phases

#### 2.3.1 Pre-intervention Phase

During which the study objective, voluntary involvement or withdrawal, and confidentiality of data were clarified to the participants, after which the participants filled out the pre-questionnaire.

#### 2.3.2 (Training Course) Interventional Phase

The training course was provided in the form of five sessions, one session every week for about 25 nurses. It was composed of two main parts: first, the infection control-training course that was based on WHO course materials on prevention, response, and control of COVID- 19 and the second was stress coping strategies during COVID-19 that were based on WHO materials on mental health [21].

#### 2.3.3 Post-interventional Phase

Three months after the intervention, the participants were asked to answer the post-intervention questionnaires.

### 2.4 Data Collection Instruments

The five main elements of the paper-based, self-administered, Arabic questionnaire (before and after) were as follow:

#### 2.4.1 First Part: Sociodemographic

Occupational, and health-related questions, this section included 15 questions about age, sex, residence, marital status, number of family members, level of education, occupation, the ward they work in, years of experience in the field, presence of chronic diseases, and history of COVID-19 infection.

#### 2.4.2 The Second Part

The Stress scale, which is part of a short version of the depression, anxiety, and stress scale (DASS) 21. The DASS-21 is a subjective and reliable tool with strong internal consistency, composed of 21 items that assess three factors: depression, anxiety, and stress. Each subscale contains seven items that were graded on a four-point Likert scale ranging from zero (not at all/never) to three (very much/always). The stress level was classified as normal (0–7), mild (8–9), moderate (10–12), severe (13–17), and extremely severe (+17), where higher scores indicate more severe emotional distress [22, 23]. It has been translated into numerous languages, making it usable by various ethnic groups. We used the validated Arabic version of the stress questionnaire [24, 25].

#### 2.4.3 The Third Part

“Fear of COVID-19 (FCV-19S) ” was composed of seven questions. Nurses’ levels of agreement were measured using a five-item Likert scale. The minimum score possible for each question is one, the maximum is five, and the total score ranged from seven to 35. The level of fear was classified as a low level of fear (score 7–21) and a high level of fear (score 22–35) [25, 26].

We used the Arabic version of the FCV-19S questionnaire. It was tested by a Saudi study using an online survey. Internal consistency was satisfactory (= .88), and concurrent validity was strong (r = .66). Therefore, the Arabic version of the FCV-19S is psychometrically robust and can be used in research studies [27].

#### 2.4.4 Fourth Part

Coping strategies, which were measured using the validated, reliable, and pretested Arabic abbreviated version of the COPE Inventory. The Brief COPE is a 28-item scale, measuring the ways that individuals use to cope with stress in their lives, and it consisted of 14 domains (each domain consisted of two items), where responses ranged from 1 (I haven't been doing this at all) to 4 (I've been doing this a lot). The scale had good internal consistency with Cronbach’s alpha of 0.83 [28, 29]. The Arabic version of the Brief COPE scale is useful in both clinical practice and research studies [30].

### 2.5 Data Management

The collected data were coded and analyzed using the Statistical Package for the Social Sciences (SPSS) version 25.0. Qualitative variables were summarized using numbers and percentages, while quantitative variables were summarized using mean ± SD. A paired t-test was applied for the paired data, and the Student t-test was used for comparing the two means. Regression analysis was used to detect the predictors. Differences were considered statistically significant when the P-value was less than or equal to 0.05.

## 3 RESULTS

The mean ages of the studied nurses were 36.70 ± 9.50. 59.2% of them were 20–39 years old; about half of them were males (52.8%). Two-thirds graduated from nursing school. About 58.4% were married, and most of them (96%) were with families that were less than four members. About one-third (32.0%) had work experience of more than 20 years and 50% were working in the paediatrics department. Before the intervention phase, 65.6% of the nurses were infected with COVID-19, and 72.8% of them received the COVID-19 vaccine. About 64% of the participants were not worried about COVID-19 vaccine side effects. About half of the studied nurses had severe levels of stress (47.2%) while the majority of them had a high level of fear of covid-19 (82.4%) before the intervention (Table **1**).

### 3.1 The Fear Score

#### 3.1.1 Pre-intervention

The mean fear scores among the studied group were 25.56 ± 6.13. There was a statistically significant difference (p = 0.01) in the mean fear score between age groups, with a higher fear score (27.25 ± 4.89) among older age groups (≥ 40 years). A highly significant difference (p = 0.002) was present in the mean fear score concerning the number of family members with a higher score among those who had fewer than four family members (25.90 ± 5.91). Also, a highly statistically significant difference (p = 0.002) in the mean fear score was found between groups working in different hospital departments, with the highest fear score present among those working in the pediatric department (27.14 ± 5.54) followed by those working in care units (25.45 ± 6.60). Moreover, a statistically significant difference (p = 0.03) in the history of COVID-19 infection criterion was associated with a higher mean fear score (27.11 ± 7.20) among those who had never had a COVID-19 infection before (Table **1**).

#### 3.1.2 Post-intervention

There was a reduction in the mean fear score in the post-intervention evaluation phase in relation to sociodemographic, work-related, and health-related factors. A non-significant reduction in the mean fear score was found in relation to age, sex, and residence groups after the intervention. A statistically significant (p = 0.01) reduction in mean fear score was found among married and separated and divorced and widowed participants (11.643 ± 2.567) and (11.551 ± 1.992), respectively. A statistically highly significant (p = 0.002) lower mean fear score was found among participants who had 1–4 family members (11.78 ± 2.34). In addition, there was a reduction in the mean fear score among nurses from different departments with a high score among those from medical departments (12.50 ± 2.54) and care units (12.18 ± 2.85). A higher fear score was observed (p = 0.000) among those who had worries about the COVID-19 vaccine (12.97 ± 2.29) (Table **1**).

#### 3.1.3 Predictors of Fear of COVID-19

Experience in years, type of department, and the presence of worries about the vaccine side were the predictors of fear of COVID-19 (P =.001, .008, and .000), respectively (Table **2**).

### 3.2 The Stress Score

#### 3.2.1 Pre-intervention

The mean stress score among the studied group was 15.27 ± 5.47. There was a statistically highly significant difference (p = 0.000) in stress mean score regarding age with a higher score (17.29 ± 4.69) (higher stress) among those ≥ 40 years of age. Also, a statistically significant (p = 0.01) difference in stress scores regarding experience was found, with a higher mean stress score among those who had more than 20 years of experience (17.45 ± 5.72) indicating higher stress levels among this group (Table **3**).

#### 3.2.2 Post-intervention

Although not statistically significant, a reduction in the mean stress score among all sociodemographic, work, and health-related factors were observed, indicating lower stress levels after the intervention. Higher scores were statistically significant (p = 0.000) among those aged 20–39 years (4.90 ± 2.34) and those who had more than four family members (7.40 ± .89) (Table **3**).

### 3.3 Pre-intervention Difference in Fear and Stress Scores

A high statistically significant reduction (p =.000) in the mean stress score was found after the intervention, decreasing from 15.27 ± 5.47 to 4.87 ± 2.14. Also, a high statistically significant reduction (p =.000) in the mean fear of COVID-19 score was evident after the intervention from 25.56 ± 6.13 to 11.92 ± 2.43, which indicates reduced fear levels (Table **4**).

### 3.4 Coping Strategies

The most applied coping strategies among nurses before the intervention were self-distraction (5.03 ± 1.53), followed by behavioural disengagement (4.36 ± 1.80), religion (4.31 ± 2.01), self-blaming (4.24 ± 1.77), and, finally, denial (3.00 ± .72). After the intervention, the most used coping strategies were religion **(**6.12 ± 1.17), followed by positive reframing (5.89 ± 1.21), acceptance (5.80 ± 1.17), venting 5.72 ± 1.26, behavioral disengagement (5.71 ± 1.37), planning (5.40 ± 1.50), and active coping (5.13 ± 1.34). The least commonly used strategy was substance abuse (2.00 ± .00) (Table **5**).

## 4 DISCUSSION

The COVID-19 pandemic has put a great strain on healthcare providers' physical and mental well-being, especially nurses. Psychological problems, such as stress, anxiety, depression, and COVID-19 dread, have been increasingly common, hurting people's social life, job performance, and patients’ safety. As a result, coping with stress programs for Healthcare Workers (HCWs) is urgently needed to promote mental health, reduce stress, and minimize the amount of anxiety, which was the study's major goal [31].

### 4.1 The Fear Score

Before the intervention, the mean ± SD of the fear score among the participants in the current study was 25.56 ± 6.13. This is lower than what recent research in the Philippines (19.6 ± 6.12) [32], Saudi Arabia (19.7 ± 7.03) [33], Jordan (23.6 ± 10.8), and Vietnam (16.7 ± 5.3) [34] had found. The observed high level of fear in this study may be attributed to the novelty of the COVID-19 pandemic-which negatively affects the level of preparedness and the rapid surges of cases and deaths in Egypt. In addition, nurses who are dealing with COVID-19 cases are exposed to a higher risk of infection.

This study found a significantly higher level of fear, and the mean ± SD of the fear score was (27.25 ± 4.89) among older age groups of 40 years and above. This comes in accordance with a Jordanian study, which reported a significantly higher level of fear and psychological distress among older HCWs. This can be attributed to the fact that older age nurses may have underlying chronic health conditions exposing them to a high risk of infection, complications, and mortality, in addition to worries related to the transmission of infection to extended family members [35].

### 4.2 The Stress Score

In the present study, the mean ± SD stress score was high (15.27 ± 5.47) among the studied group, and about 84% of them had a certain level of stress. This conforms with three studies in Egypt [36], which found a significant prevalence of specific mental health problems related to perceived stress, which was 98.5% among HCWs. Another study found a high prevalence of stress, which was 80.9% among HCWs [20], [37]. Also [38], a cross-sectional study, which was conducted in five COVID-19 isolation governmental Egyptian hospitals, reported that 52.1% of the studied nurses had a moderate level of stress by the Nursing Stress Scale (NSS).

We reported higher stress scores mean ± SD (17.29 ± 4.69) (higher stress level) among those of 40 years of age, which were statistically significant (p = 0.01). Higher stress means the score was found among those with more than 20 years of clinical experience (over 20 years) (17.45 ± 5.72). These results come in agreement with other studies, which reported that participants aged over 40 years showed statistically higher levels of fear and stress when compared with participants aged over 40 years (p < 0.001 and p < 0.001, respectively), and those with more clinical experience (over 20 years) showed statistically higher levels of fear and stress (p < 0.001 and p < 0.001, respectively) [39].

These results may be attributed to the fact that older nurses are more susceptible to infection, adverse events, and deaths, and those with more experience are usually older and exposed to higher workloads, responsibilities, and consequent stress. Additionally, during the pandemic, an overuse and misuse of the internet led to the development of a preliminary model of the neurological basis of Internet addiction. According to Caplan, online communication allows one to avoid negative feelings like loneliness and anxiety. Similar to many addictions, the reward system's activation can raise dopamine levels in this system, generating a dependent framework for excessive internet use [40].

In contrast to these findings, an Egyptian study on physicians found significantly higher levels of emotional exhaustion among younger physicians with work experience of fewer than ten years [41]. These inconsistencies may be attributed to the fact that younger generations had fewer strategies to cope with the stressors and interactions with the work environment, and the older generation was exposed to a higher risk of infection and work responsibilities, making them more adept at coping with stress.

This study found that experience in years, type of department, and presence of worries about vaccine side effects were the predictors of fear of COVID-19 (P = .001, .008, and .000, respectively). This finding comes in line with an Egyptian study among physicians, which reported that dealing with critical cases and cases of COVID-19 were among the predictors of burnout among the studied group [41, 42]. In addition, worries about vaccine side effects were among the predictors of fear of COVID-19 because no prior experience or successes with such an approach had been reported in the past. The possibility of side effects is supported by a CDC survey that declared only 63% of HCWs are on board with being vaccinated right away. No vaccine developed in such a short time frame can be truly safe. Moreover, without sufficient years of trials, it is difficult to predict their long-term side effects [43].

### 4.3 Pre-intervention Difference in Fear and Stress Scores

The current study found a statistically highly significant difference between the mean stress and fear scores before the intervention and after it, with a significant reduction of the mean stress and fear scores in the post-intervention phase. This result coincides with that of Wu *et al*. (2020) [44], where nurses who received COVID-19 epidemic training reported a significant reduction in apprehension about the disease and increased mental health functioning compared with those nurses who had not received training related to the management of COVID-19. In addition, RN *et al*., stated that resilience-improving psychological support is essential to preserve the mental health of nurses and help relieve stress and anxiety [45]. Additionally, another study stated that nurses who reported no COVID-19-related training experienced higher levels of fear of COVID-19.

In the present study, after the intervention that reduced the level of fear and stress among the studied nurses, there was a shift in the used coping strategies toward active coping. The most common coping strategies were religion **(**6.12 ± 1.17), followed by positive reframing (5.89 ± 1.21), acceptance (5.80 ± 1.17), venting (5.72 ± 1.26), behavioral disengagement (5.71 ± 1.37), planning (5.40 ± 1.50), and active coping (5.13 ± 1.34), Similarly, Girma *et al*., (2021) stated that active coping, denial, behavioral disengagement, self-blame, and religious coping strategies positively predicted the COVID-19-related stress score [46].

### 4.4 Coping Strategies

Coping during the pandemic is challenging in many ways due to the rapid spread of the disease, the large number of cases and deaths, mistrust in the available interventions and health system, ignorance, and misinformation, but it is important to ensure physical and emotional coping and resilience to preserve nurses' mental health [47].

Before the intervention in the current study, the most used coping strategies were self-distraction (5.03 ± 1.53) and behavioral disengagement (4.36 ± 1.80). Similarly, studies show that nursing students’ use of avoidance and transference as coping strategies increased during the COVID-19 pandemic [48]. During the third wave of the COVID-19 pandemic, Egyptian university students used the following stress-reduction techniques: The most popular coping strategies were isolation and excessive sleeping, even if these coping strategies might be signs of stress [46].

The most used coping strategy adopted by the studied nurses after the intervention was religion. This comes in accordance with a Saudi study in which coping strategies were assessed using the Brief Resilient Coping Scale (BRCS) by Al Solaris *et al*., (2021) in their cross-sectional online survey, which was conducted on Saudi student nurses. Religion was reported as the most frequently used coping strategy to assess the perceptions of risk, fear, mental health status, and coping strategies among Saudi student nurses [49, 50].

### 4.5 Strengths

this study recruited a representative number of nurses from all hospital departments. Moreover, to our knowledge, this was the first intervention study assessing the effect of coping with stress sessions on fear of COVID, stress, and coping strategies among nurses in Egypt, where almost all available studies are cross-sectional studies.

## CONCLUSION

According to the findings of this study, almost half of the evaluated nurses had significant levels of stress. Almost half of the studied nurses had severe levels of stress while the majority of them had a high level of fear of COVID-19. After the intervention, the stress and fear scores were reduced by half or even less. Older age, longer work experience, and worries about the vaccine were the predictors of fear of COVID-19. The coping strategies used after the intervention shifted toward active coping strategies.

## RECOMMENDATIONS

The previous findings point to the actual need for 1) Screening for mental health disorders among healthcare providers, targeting vulnerable groups and high-risk healthcare providers; 2) The development and implementation of mental health promotion and coping programs in hospitals, particularly those dealing with COVID-19; 3) Implementation of specific case management and follow-up systems and 4) The effect of internet addiction and excessive social networking use could be unnoticed and underestimated, so further studies should be conducted among different age groups and different occupations

## Figures and Tables

**Table 1:** Participants characteristics and its relationship with Fear score pre and post intervention.

**Variables**		**N**	**%**	**Fear Score**	
				**Pre-intervention** 25.56±6.13 **Mean± SD**	**Post -intervention** 11.92±2.43 **Mean± SD**
**Age** (**Mean± SD)** 36.70±9.50 **p**	**20-<40** **≥40**	74 51	59.2% 40.8%	24.39±6.63 27.25±4.89 .01**	12.18±2.58 11.52±2.15 .13
**Sex** **P**	**Male** **Female**	66 59	52.8% 47.2%	25.10±6.29 26.06±5.95 .38	11.590±2.52 12.28±2.28 .11
**Residence** **P**	**Urban** **rural**	29 96	23.2 76.8	23.68±6.36 26.12±5.97 .06	11.79±2.84 11.95±2.31 .75
**Occupation** **P**	**Nurse** **supervisor**	36 89	28.8 71.2	26.41±5.52 25.21±6.35 .322	11.69±2.60 12.01±2.37 .512
**Marital Status** **p**	Single Married others(separated-divorced-widowed)	23 73 29	18.4% 58.4% 23.2%	26.34±7.46 25.38±5.82 25.37±5.89 .79	13.26±2.11 11.64±2.56 11.55±1.99 .01**
**Family Members** **P**	1-4 More than 4	120 5	96.0% 4.0%	25.90±5.91 17.40±6.22 .002**	11.78±2.34 15.20±2.38 .002**
**Years of Experience** **p**	<5 5- <10 years 10- <15 years 15- <20 ≥20	27 19 26 13 40	21.6% 15.2% 20.8% 10.4% 32.0%	24.66±6.43 25.31±5.14 23.65±6.97 26.46±4.57 27.22±5.99 .174	12.14±2.47 12.42±2.69 12.73±2.49 11.69±2.01 11.07±2.20 .062
**Department Type** **P**	Care units Emergency depart Medical department Paediatric department Others(infection control)	11 20 20 63 11	8.8% 16.0% .8% 16.0% 50.4% 8.0%	25.45±6.60 24.70±5.50 24.85±6.27 27.14±5.54 19.45±6.29 .002**	12.18±2.85 11.55±2.37 12.50±2.544 11.47±2.21 13.81±2.40 .030*
**Got COVID 19** **p**	Yes No	82 43	65.6% 34.4%	24.74±5.35 27.11±7.20 .039*	11.792±2.51 12.16±2.27 .422
**Had Worries about the Vaccine** **p**	Yes No	45 80	36.0% 64.0%	25.46±7.40 25.61±5.33 .89	12.97±2.29 11.32±2.32 .000**
Up took COVID 19 vaccine (post intervention)	Yes No	91 34	72.8% 27.2%		

**Note**: * Significance at 0.05.

**Significance at 0.01.

**Table 2:** Multiple linear regression analysis for the predictors of fear of COVID 19 (post intervention).

**Model**	**B**	**S.E.**	**Beta**	**t**	**Sig.**
**Marital Status**	-.577-	.311	-.153-	-1.853-	.066
**Experience in years**	-.488-	.148	-.310-	-3.297-	.001**
**Department type**	.383	.142	.257	2.694	.008**
**Had worries about vaccine side effects**	-1.582-	.417	-.313-	-3.794-	.000**

* Significance at 0.05.

**Significance at 0.01

**Table 3:** Stress score pre and post intervention in relation to participant characteristics.

-		**Stress Score**	
**Variables**		**Pre-intervention** 15.27±5.47 **Mean± SD**	**Post-intervention** 4.87±2.14 **Mean± SD**
**Age** **p**	**20-<40** **≥40**	13.87±5.56 17.29±4.69 .000**	4.90±2.34 4.82±1.82 .000**
**Sex** **p**	**male** **female**	14.83±5.83 15.76±5.03 .34	4.92±2.00 4.81±2.30 .77
**Residence**	**urban** **rural**	13.89±6.04 15.68±5.24 .12	5.24±2.48 4.76±2.02 .29
**Occupation**	**Nurse** **Supervisor**	16.19±5.15 14.89±5.57 .23	5.33±1.78 4.68±2.24 .12
**Night shifts**	**0.00- 2** **≥3**	15.31±5.50459 14.00±4.76 .63	4.84±2.16 5.75±.95 .40
**Family members** **p**	**1-4** **more than 4**	15.45±5.34 10.80±7.25 .06	4.76±2.11 7.40±.89 .007**
**Chronic disease** **P**	**Yes** **No**	16.76±5.27 14.80±5.47 .08	4.83±2.13 4.88±2.15 .91
**Experience**	**<5** **5- <10 years** **10- <15 years** **15- <20** **≥20**	14.74±5.95 15.52±4.18 12.84±5.13 14.15±3.71 17.45±5.72 .013*	5.07±2.20 4.57±2.29 4.96±2.52 4.53±2.10 4.92±1.83 .915
**Department type** **P**	**Care units** **Emergency d Department** **Medical Department** **Paediatric Department** **others (Infection Control)**	15.45±5.85 15.15±5.05 12.85±5.11 16.65±5.12 12.40±6.25 .01**	5.27±2.86 4.25±1.11 5.05±2.74 4.74±2.02 6.10±2.07 .33

**Note**: * Significance at 0.05.

**Significance at 0.01

**Table 4:** the difference between stress and fear of COVID 19 scores pre and post intervention.

-	-	**Pre-intervention**		**Post –intervention**		**Paired t (p)**
Stress score#	**Mean± SD**	15.27±5.47		4.87±2.14		18.79 (.000)**
Levels of stress	Normal Moderate Severe Extremely severe	20 11 59 35	16.0% 8.8% 47.2% 28.0%			
Fear score ##	**Mean± SD**	25.56±6.13		11.92±2.43		18.79 (.000)**
Levels of fear of COVID 19	Low level High level	22 103	17.6% 82.4%			

**Note**: # Mean difference (Mean± SD) = 10.40±6.18.

## Mean difference (Mean± SD) = 13.64±7.08.

**Table 5:** Coping strategies adopted by the studied group pre and post intervention.

**Coping Subscales**	**Pre intervention (Mean± SD)**	**Post intervention (Mean± SD)**	**Mean Difference Mean± SD**	**Paired t Test**	**P value**
Humor	3.88±1.44	4.12±1.37	.24±2.03	1.36	.175^⁎⁎^
Religion	4.31±2.01	6.12±1.17	1.81±2.48	8.17	.000**
Acceptance	3.57±1.79	5.80±1.17	2.22±2.05	12.12	.000**
Instrumental support	3.89±2.02	3.80±1.39	-.09-±2.50	-.42-	.669
Planning	3.18±1.30	5.40±1.50	2.21±2.03	12.17	.000**
Self- blaming	4.24±1.77	3.88±1.17	-.35-±1.94	-2.02-	.045*
Positive- reframing	3.36±1.73	5.89±1.21	2.53±2.17	13.05	.000**
Venting	4.14±1.65	5.72±1.26	1.57±1.91	9.22	.000**
Behavioural disengagement	4.36±1.80	5.71±1.37	1.344±2.18	6.86	.000**
Emotional support	3.69±1.65	5.13±1.15	1.44±2.15	7.47	.000**
Substance use	3.89±.99	2.00±.00	-1.89-±.99	-21.22-	.000**
Denial	3.00±.72	4.11±1.24	1.10±1.53	8.021	.000**
Active- coping	3.28±1.01	5.13±1.34	1.85±1.64	12.61	.000**
Self-distraction	5.03±1.53	3.17±.76	-1.85-±1.72	-12.02-	.000**

**Note**: **Significance at 0.01

## Data Availability

All data that support the research finding is available from the corresponding author [S.A.A] upon request from the editor.

## LIST OF ABBREVIATIONS

BRCS: Brief Resilient Coping Scale
**COVID-19**: The coronavirus 2019 disease outbreak
DASS: Depression, anxiety and stress scale **(**)
FCV-19S: Fear of COVID-19 scale
HCWs: Health care workers
NSS: Nursing Stress Scale
**SARS-CoV-2**: Severe acute respiratory syndrome coronavirus 2
SPS: Statistical Package for the Social Sciences
WHO: World Health Organization
